# The terroir of Tempeh: Strong region-specific signatures in the bacterial community structures across Indonesia

**DOI:** 10.1016/j.crmicr.2024.100287

**Published:** 2024-10-10

**Authors:** Wisnu Adi Wicaksono, Oluwakemi Elizabeth Akinyemi, Birgit Wassermann, Samuel Bickel, Antonius Suwanto, Gabriele Berg

**Affiliations:** a Institute of Environmental Biotechnology, Graz University of Technology, Graz, Austria; b Department of Biology, Faculty of Mathematics and Natural Science, IPB University, Bogor, Indonesia; c Leibniz-Institute for Agricultural Engineering and Bioeconomy Potsdam (ATB), Potsdam, Germany; d Institute for Biochemistry and Biology, University of Potsdam, Potsdam, Germany

**Keywords:** Fermented food, Tempeh, Terroir, Microbiome, Amplicon sequencing, Lactobacilli

## Abstract

Tempeh, a soybean product from Indonesia, is created through fermentation by *Rhizopus* spp. and associated bacteria. Here, we aim to get an overview of the variability of the tempeh microbiota across Indonesia and disentangle influencing factors. We found high variability in bacterial abundance (10^3^ – 10^9^ copies g^-1^), richness (n_ASV_ = 40 – 175 ASVs), and diversity (H’ = 0.9 – 3.5) in tempeh. The primary factor affecting this variation was the region, where the tempeh was produced. Interestingly, tempeh samples obtained from geographically close areas tended to share similar bacterial profiles, suggesting a "terroir" of tempeh. Additionally, tempeh wrapped in banana leaves had a higher abundance of enterobacteria in comparison to tempeh wrapped in plastic but also tended to have a higher total bacterial and lactobacilli abundance. Despite all variability, the tempeh core microbiome consists *Lactobacillales* and *Enterobacteriales.* This study demonstrates a high variability of bacterial diversity in traditional tempeh from local producers highlighting a strong regional influence across Indonesia.

## Highlights

• A characteristic "terroir" associated with the structures of bacterial communities.
• Impact of packing material on the bacterial diversity and community structures.
• *Lactobacillus* and *Enterobacter* were the predominant bacterial taxa.
• Packaging with banana leaves promotes the growth of beneficial microorganisms.

## Graphical abstract

**Figure:**
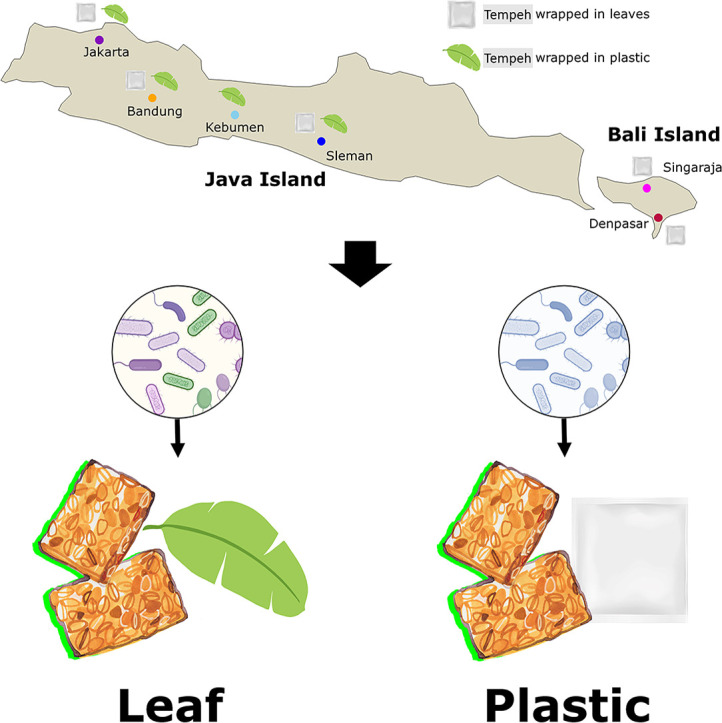

## Introduction

Fermented foods, which were originally developed as a means of preserving food substrates, are important parts of traditional food culture and have been part of the human diet all over the world since early civilization (Mukherjee et al., 2023). Incorporating fermented foods into our diet offers a simple and natural approach to promoting overall health and well-being. The health benefits of the consumption of fermented foods i.e., anti-oxidant, anti-microbial, anti-inflammatory, and anti-diabetic result from the activity of diverse living microorganisms, such as bacteria and yeasts, with probiotic functions (Marco et al., 2017; McGovern et al., 2004). Fermented foods and their microbiome can help restore and maintain a healthy balance of gut bacteria, leading to better digestion, nutrient absorption, and immune function (Diez-Ozaeta and Astiazaran, 2022). Fermented foods have been linked to a decreased risk of various gastrointestinal disorders, such as irritable bowel syndrome and inflammatory bowel diseases (Marco et al., 2017). Additionally, the diverse microbes found in fermented foods help prevent the growth of pathogenic microorganisms (Skowron et al., 2022). Therefore, gaining a thorough understanding of microbial diversity and community structures is essential to ensure the health benefits of fermented foods.

Fermented food is traditionally highly diverse and heterogenous, but several common factors are known that influence its quality and characteristics. While many products are still fermented by indigenous microbial communities, different starter cultures can have varying effects on the final product (Tamang et al., 2016). Furthermore, the selection of fermentation conditions including nutrient content, pH levels, temperature, oxygen, and the duration of fermentation play a role in affecting the fermentation process and the quality of fermented products (Xiang et al., 2019). The type and quality of the raw materials used can influence microbial diversity and metabolic activity throughout the fermentation process (Kårlund et al., 2020; Li et al., 2015; Yue et al., 2021). Also, traditional fermentation methods and industrial processing can have an impact on the properties of the final product (Crafack et al., 2014; De Vuyst et al., 2023; Yue et al., 2021). The dynamic interplay of these factors underscores the complexity of fermented food production and its sensitivity to both raw material characteristics and process conditions. The versatility of microbial communities is one of the major characteristics of fermented food; however, this factor is poorly understood in terms of health benefits and biosafety aspects.

Tempeh, a fermented soybean product that originated in Indonesia, has gained significant attention worldwide and popularity in recent years due to its nutritional advantages and distinct flavor. The manufacturing of tempeh primarily involves cottage industries or home industries. There are approximately 81,000 tempeh producers in Indonesia who collectively manufacture 2.4 million tons of tempeh on an annual basis (Badan Standarisasi Nasional, 2012). The demand for tempeh is expected to reach $5.8 billion (USD) by 2026, with a compound annual growth rate of 6.1 percent (Shanti et al., 2023). The Asia Pacific region is projected to maintain its position as the largest market until 2030, followed by Europe. Additionally, the tempeh market in North America is predicted to experience substantial growth in the next decade.

The production of tempeh entails a specialized fermentation process, in which soybeans are inoculated with a starter culture that contains either *Rhizopus oligosporus* or *R. microsporus* var. *oligosporus* (Ahnan‐Winarno et al., 2021). This fermentation process leads to the formation of a firm texture and a wide range of flavors, making tempeh a versatile ingredient for use in various culinary contexts. The microbiome of tempeh is dominated by the fungal strain used for fermentation (*Rhizopus* spp.); however, production processes and the production environment substantially influence the diversity and composition of rare microbiota, affecting sensory characteristics and nutritional composition (Ahnan‐Winarno et al., 2021; Barus et al., 2008; Erdiansyah et al., 2021). However, certain bacterial taxa within the Enterobacteriaceae family, such as *Klebsiella* and *Citrobacter*, are responsible for the production of vitamin B complex in tempeh (Keuth and Bisping, 1994). Previous research has predominantly focused on how tempeh production methods, such as starter cultures and preparation processes, affect the tempeh's microorganisms (Nurdini et al., 2015; Pramudito et al., 2021). However, an overview about variability of bacterial community structures of tempeh is still missing.

Here we performed qPCR and high-throughput amplicon sequencing to investigate the bacterial abundance and community structures of tempeh from multiple cities and provinces in Indonesia. We aimed to address the following questions: (1) does the microbiome of tempeh vary between different production locations? (2) does it vary based on the type of packaging used? And (3), if disparities exist, which particular microbial taxa are either enriched or lacking in these products? We hypothesized that local producers and packaging materials will have a significant impact on bacterial abundance and community structure in final tempeh products. Through this research, we aim to shed light on the less explored aspects of tempeh production and offer a more comprehensive understanding of the factors that influence its microbial composition.

## Materials and methods

### Experimental design and sample processing

**Fig. 1:**
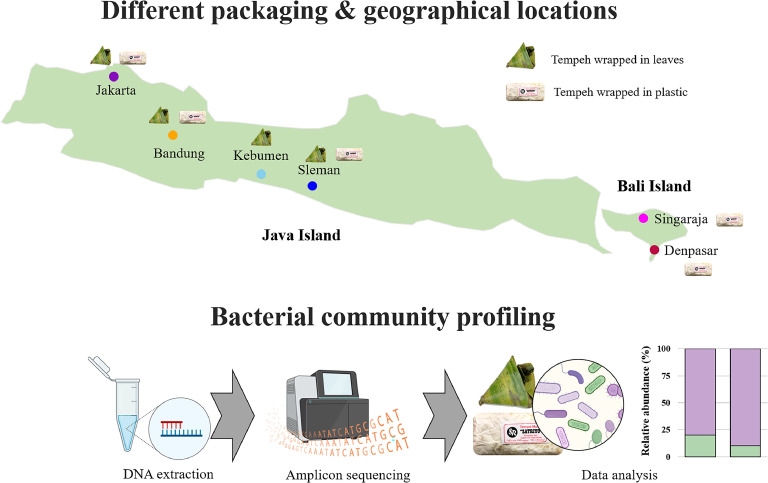
Summary of sampling location and the conducted microbial community analyses.

We obtained tempeh samples (*n* = 48, Fig. 1, Supplementary Table S1) from 18 traditional markets from six cities and four provinces i.e., Bandung (West Java), Jakarta (DKI Jakarta), Sleman (Jogjakarta), Kebumen (Central Java), Denpasar and Singaraja (Bali). In total, we obtained 18 tempeh samples wrapped in banana leaves and 30 tempeh samples wrapped in plastic. Only 3 cities i.e., Bandung, Sleman, and Jakarta had both tempeh wrapped in banana leaves and plastic. From Denpasar and Singaraja, we only obtained tempeh wrapped in plastic whereas from Kebumen, only tempeh samples wrapped in leaves were obtained. Each tempeh sample was sliced into small pieces and stored in DNA /RNA Shield™ (Zymo Research, Freiburg, Germany) at −20°C. All samples underwent DNA extraction following 10 days of storage under the storage conditions.

Tempeh samples (10 g) were homogenized in 10 mL sterile NaCl (0.85 %) solution using a Stomacher lab blender (BagMixer, Interscience, Saint-Nom-la-Bretèche, France) for three minutes before DNA extraction. A total of 2 mL of processed and homogenized tempeh were centrifuged for 20 min at 16,000 g and pellets were used for DNA extraction. Total DNA was extracted using FastDNA SPIN Kit for Soil and the FastPrep Instrument (MP Biomedicals, Santa Ana, CA, USA) according to the manufacturer's protocol and kept at −20 °C until PCR reactions were carried out.

### Bacterial quantification in tempeh

We used quantitative real-time PCR (qPCR) based on SYBR Green fluorescence to calculate microbial abundance in the tempeh samples (copies marker genes/gram). We used primer set 515f- 806r (Caporaso et al., 2011), F-lac/R-lac (Walter et al., 2001), F‑*ent*/R‑*ent* (Castillo et al., 2006; Leser et al., 2002; Sghir et al., 2000) to calculate the absolute total bacterial, lactobacilli and enterobacteria abundance, respectively. Fluorescence quantification was carried out using the Rotor-Gene 6000 real-time rotary analyzer (Corbett Research, Sydney, Australia) with initial denaturing at 95 °C for 10 min, followed by 40 cycles of denaturing at 95 °C for 30 s, annealing at 54 °C (for total bacteria) or 60 °C (for lactobacilli) or 63 °C (for enterobacteria) for 30 s, and extension at 72 °C for 30 s, and a final melting curve.

### 16S rRNA gene amplicon sequencing

The 16S rRNA gene V4 and V5 hypervariable region were amplified from the total DNA using the primer pair 515f-806r (Caporaso et al., 2011). The primers contained Illumina indexes (barcode sequences) for multiplexing. Two technical replicates were used for each sample. To prevent the host plastic and mitochondrial DNA from being amplified by PCR, peptide nucleic acid (PNA) clamps were applied (Lundberg et al., 2013). Polymerase chain reactions (PCRs) were carried out in Whatman Biometra® Tpersonal thermocycler (Biometra 141 GmbH, Göttingen, Germany) with 35 cycles at 94 °C for denaturation for 45 s, 54 °C annealing for 60 s, and 72 °C elongation for 90 s. To confirm successful amplification, PCR products were visualized on 1 % agarose gels and subsequently purified using the Wizard® SV Gel and PCR Clean-Up kit (Promega). The sequencing of the barcoded amplicons was carried out on an Illumina MiSeq (2 × 250 bp paired-end reads) by the sequencing provider Genewiz (Leipzig, Germany). Amplicon sequences were deposited at the European Nucleotide Archive (ENA) under the project number PRJEB75498.

### Bioinformatics and statistical analysis

Cutadapt was used to demultiplex the data and remove primer sequences. In QIIME2 (Bolyen et al., 2019), the DADA2 algorithm (Callahan et al., 2016) was used to quality filter, denoise, and eliminate chimeric sequences. The resulting representative sequences, known as amplicon sequences variants (ASVs), were further classified using the VSEARCH algorithm against the SILVA v138 database (Quast et al., 2012; Rognes et al., 2016). ASV tables and taxonomic classifications were then used as input for bacterial community analysis. Sequences that were classified as chloroplast and mitochondrial DNA as well as other non-bacteria reads were removed prior to further analysis. Amplicon sequencing generated 3416273 bacterial sequences that were assigned to 839 bacterial ASVs. The core microbiome, which was defined as ASVs with an occurrence in 90 % of the total samples, was identified. Sequences of ASVs that were identified as components of the core microbiome were aligned using MUSCLE for phylogenetic analyses (Edgar, 2004), and the distance matrices were calculated with the maximum-likelihood algorithm in MEGA X (Molecular Evolutionary Genetic Analysis) (Kumar et al., 2018)

Except when otherwise noted, statistical analysis and graph rendering were done in R studio version 2021.09.0 [26]. The non-parametric Kruskal-Wallis test was used to identify significant differences (*P* < 0.05) in bacterial gene copy counts per gram of fresh weight samples, bacterial richness, and diversity between different groups. Dunn's test of multiple comparisons was used to compare the groups, and the Benjamini-Hochberg technique was used to correct the P values. Before the alpha and beta diversity analysis, the bacterial community dataset was normalized to the lowest number of read counts by randomly choosing subsets of sequences (13,436 reads). According to the respective rarefaction curves, the sampling size was enough to capture overall bacterial richness (Supplementary Fig. S1). Taxonomical compositions at the genus level were visualized using a bar plot. Beta diversity analysis was performed using Bray-Curtis dissimilarity matrices that were generated from the normalized dataset. The dissimilarity matrix distances were further subjected to Permutational ANOVA (PERMANOVA) using the Adonis function to test for significant effects of experimental factors on the bacterial community structures. The SparCC analysis was conducted using MicrobiomeAnalyst, a web-based data analysis platform (Chong et al., 2020). The spearman correlation coefficient was utilized for calculating the correlation analysis. Additionally, Cytoscape 3.8.2 software (Saito et al., 2012) was employed for constructing and visualizing the network of tempeh microbiome.

## Results

### Impact of geographical origin on bacterial abundance and diversity in tempeh

**Fig. 2:**
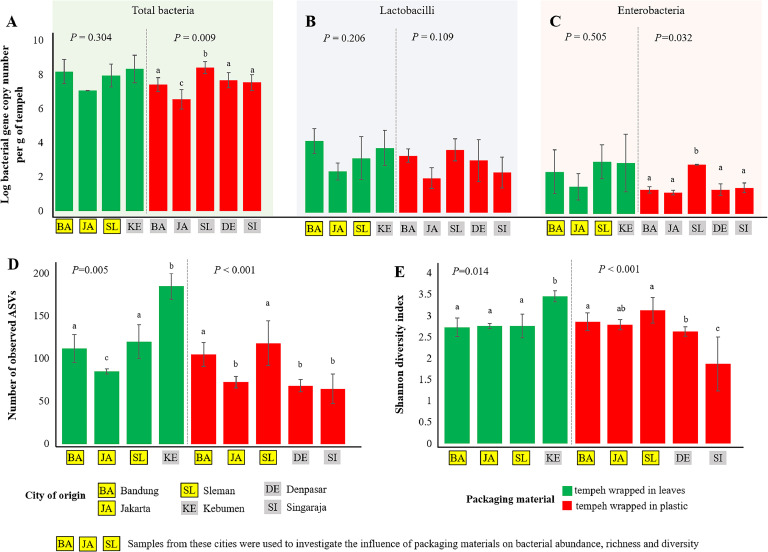
Comparison of bacterial abundance and diversity between tempeh collected from different geographical origins and with different packaging. Total bacterial (A), lactobacilli (B), and enterobacterial (C) abundance were measured using a qPCR-based method. Number of bacterial ASVs (D) and Shannon index (E) were calculated from the high-throughput amplicon sequencing.

The geographical origin significantly impacted the bacterial abundance in tempeh wrapped in plastic. For plastic-wrapped tempeh, we found a significant impact of the geographical origin on total bacterial (*P* = 0.009) and enterobacteria (*P* = 0.032) 16S rRNA gene abundances (Fig. 2A-C). There were no differences in lactobacilli 16S rRNA gene abundances between tempeh wrapped in plastic from different cities (*P* = 0.109). Tempeh wrapped in plastic from Jakarta had significantly lower total bacterial 16S rRNA gene abundances (3.5 × 10^6^ copies g^-1^) in comparison to tempeh wrapped in plastic from Bandung (2.6 × 10^7^ copies g^-1^), Denpasar (4.8 × 10^7^ copies g^-1^), Singaraja (3.5 × 10^7^ copies g^-1^) and Sleman (2.7 × 10^8^ copies g^-1^). Tempeh wrapped in plastic from Sleman had the highest enterobacteria 16S rRNA gene abundances (6.7 × 10^2^ copies g^-1^) compared to other samples. No influence of the geographical origin was observed for tempeh wrapped in banana leaves on total bacterial (*P* = 0.304), lactobacilli, (*P* = 0.206), and enterobacteria (*P* = 0.505).

To investigate the influence of packaging materials on bacterial abundance, we selected data from tempeh that were obtained from Bandung, Sleman, and Jakarta because both tempeh wrapped in banana leaves and plastic were obtained from these cities. Our analysis indicated that the abundance of enterobacteria (*P* = 0.011) was affected by packaging materials. Tempeh wrapped in leaf had a higher abundance of enterobacteria in comparison to tempeh wrapped in plastic (3.6 × 10^2^ copies g^-1^ and 4.8 × 10^1^ copies g^-1^, respectively). A trend to higher total bacterial (*P* = 0.099) and lactobacilli (*P* = 0.072) abundance was also observed in tempeh wrapped in leaves (total bacteria - 8.3 × 10^7^ copies g^-1^ and lactobacilli - 3.2 × 10^4^ copies g^-1^) in comparison to tempeh wrapped in plastic (total bacteria - 2.5 × 10^7^ copies g^-1^ and lactobacilli - 7.1 × 10^2^ copies g^-1^). We noted a strong correlation between the abundance of lactobacilli and total bacterial abundance (Supplementary Figure S2A), as well as between enterobacterial abundance and total bacterial abundance (Supplementary Figure S2B). This suggests that these groups may be key components of the bacterial community in tempeh.

Geographical origin also affected bacterial richness and diversity in both tempeh wrapped in leaves and plastics (*P* < 0.05, Fig. 2D and 2E). Tempeh wrapped in leaves from Kebumen had the highest bacterial richness (n_ASV_ = 185) and diversity (H’ = 3.46) in comparison to other tempeh wrapped in leaves (Fig. 2D and 2E). For plastic-wrapped tempeh, samples from Sleman had the highest bacterial richness (n_ASV_ = 118) and diversity (H’ = 3.13) compared to other tempeh wrapped in plastics. Interestingly, the cities Sleman and Kebumen are relatively close to each other. As only three cities (Bandung, Sleman, and Jakarta) had tempeh wrapped in both banana leaves and plastic, we utilized those samples to analyze the impact of packaging materials. Our findings revealed no significant effect of packaging materials on bacterial richness (*P* = 0.716) and diversity (*P* = 0.120).

### Impact of packaging materials on bacterial community structures

**Fig. 3:**
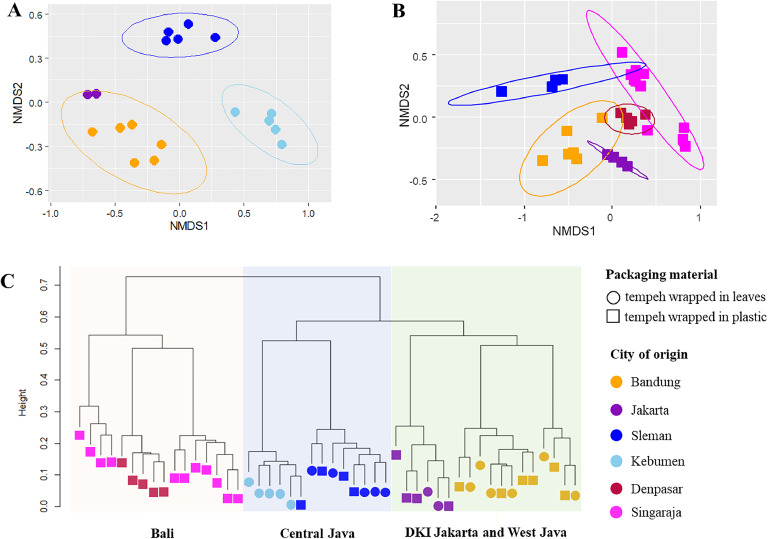
Bacterial community compositions of tempeh collected from different geographical origins and with different packaging. The nonmetric multidimensional scaling (NMDS) plots show the clustering of bacterial communities from tempeh wrapped in leaves (A) and plastic (B). Hierarchical clustering of bacterial community compositions of tempeh collected from different cities indicates clusters based on the provinces.

The geographical origin also affected bacterial community structures in final tempeh products (*P* < 0.001). Adonis analysis indicated that the geographical origin explained 76.4 % and 62.3 % of bacterial community variations in tempeh wrapped in leaves and plastic, respectively. Supporting the Adonis analysis, a non-metric multidimensional scaling (NMDS) plot depicted a strong clustering by the geographical origin in both tempeh wrapped in leaves and plastic (Fig. 3A and 3B). In contrast, using bacterial community profiles of tempeh samples that were obtained from Bandung, Sleman, and Jakarta, we did not observe a significant effect of packaging materials on bacterial community structures (*P* = 0.340 – R^2^ = 0.042). Additionally, tempeh samples that were obtained from cities that are in close proximity, tended to cluster together (Fig. 3C), such as tempeh samples from Denpasar and Singaraja (Bali cluster), samples from Jakarta and Bandung (West Java and DKI Jakarta cluster), and samples from Sleman and Kebumen (Central Java cluster).

### *Lactobacillus* and *Enterobacter* were predominant bacteria in tempeh

**Fig. 4:**
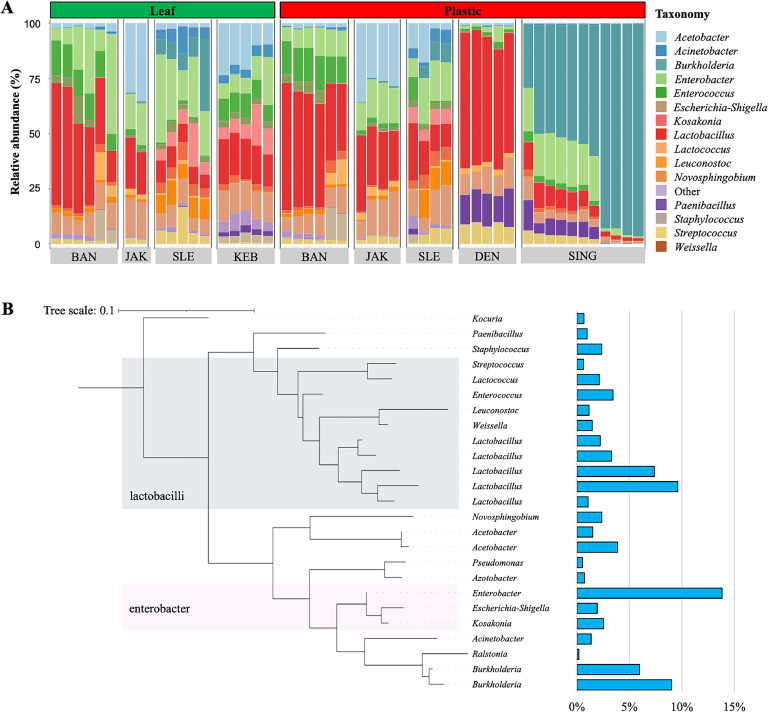
Comparative assessment of bacterial community composition and tempeh core microbiome. Bar plot represents bacterial composition at genus level (A). A phylogenetic tree was constructed with partial 16S rRNA gene of the core microbiome (B). City of origin – BAN: Bandung, JAK: Jakarta, SLE: Sleman, KEB: Kebumen, DEN: Denpasar, and SING: Singaraja.

From all tempeh samples, at the genus level, *Lactobacillus* and *Enterobacter* dominated tempeh samples contributing to overall 41.0 % and 38.5 %, respectively (Fig. 4A). The relative abundance of *Enterobacter* was higher in tempeh wrapped in leaves (18.5 %) in comparison to tempeh wrapped in plastic (11.6 %). Relative abundance of other bacterial genera i.e., *Acinetobacter, Kosakonia, Escherichia-Shigella,* and *Paenibacillus* were also higher in tempeh wrapped in leaves (3.2, 5.2, 3.1, and 2.4 %, respectively) in comparison to tempeh wrapped in plastic (0.9, 0.9, 1.2 and 0.7 %, respectively). In contrast, the relative abundance of *Staphylococcus* was higher in tempeh wrapped in plastic (4.3 %) in comparison to tempeh wrapped in leaves (0.9 %). We observed a high relative abundance of *Burkholderia-Caballeronia-Paraburkholderia*, which contributed to 65.5 % of total reads, exclusively in tempeh wrapped in plastic from Singaraja. Moreover, *Paenibacillus* was found in a high relative abundance in tempeh samples wrapped in plastic that were obtained from Denpasar and Singaraja.

The core microbiome of tempeh comprised 25 bacterial ASVs with at least 90 % prevalence. Interestingly, these ASVs contributed to a large portion (average of 79.7 %) of the total reads (Fig. 4B). Most members of the core microbiome belonged to the bacterial orders *Lactobacillales* (*n* = 10 ASVs) such as *Lactococcus, Enterococcus,* and *Lactobacillus, Enterobacteriales* (*n* = 3 ASVs) i.e., *Enterobacter, Escherichia-Shigella*, and *Kosakonia,* as well as *Burkholderiales* (*n* = 2 ASVs) i.e., *Ralstonia* and *Burkholderia*. Two ASVs belonging to *Lactobacillus* and *Enterobacter* were the dominant members of the core microbiome which contributed to an average of 9.6 % and 13.8 %, respectively.

**Fig. 5:**
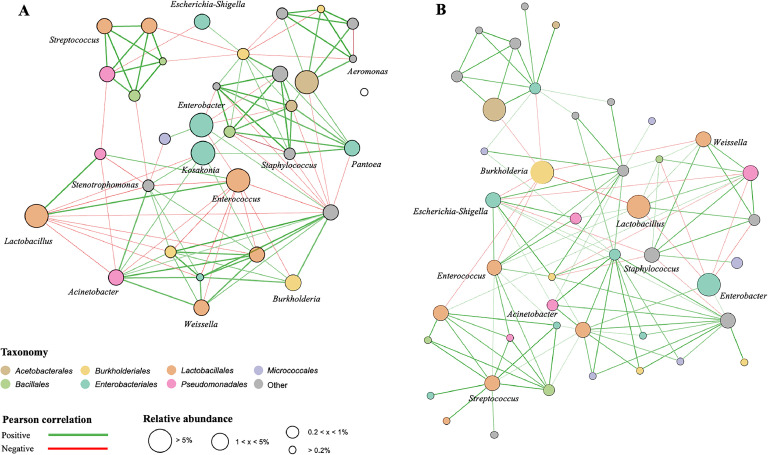
A correlation network analysis (Pearson's correlation) of bacterial communities in tempeh wrapped in leaves (A) and plastic (B). Node sizes represent the relative abundance of bacterial genera and the thickness of lines connecting nodes represents the value of Pearson's correlation coefficients.

To gain a deeper insight into the interactions among bacterial taxa in the tempeh final products, we performed a correlation network analysis based on the amplicon sequencing data of tempeh wrapped in leaves and plastic. The bacterial network of tempeh wrapped in leaves and plastic consisted of a total of 112 and 114 edges of significant correlation from 45 bacterial genera (Fig. 5A and 5B). Among them, 76 and 36 pairs of positive and negative correlations, respectively, were identified from the bacterial network of tempeh wrapped in leaves, whereas 98 and 16 pairs of positive and negative correlations, respectively, were identified from the bacterial network of tempeh wrapped in plastic.

A total of six bacterial genera demonstrated a high degree of connection with ≥ 8 edges per node, including *Lactococcus, Enterococcus, Comamonas, Lactobacillus*, and *Staphylococcus*. Specific attention is given to the interaction between putative beneficial and pathogenic taxa. In tempeh wrapped in leaves, a potentially beneficial taxon namely *Lactobacillus* was negatively correlated to putatively pathogenic *Stenotrophomonas* and *Acinetobacter* as well as *Kosakonia*. In contrast, *Lactobacillus* had a positive correlation with *Azotobacter* and *Enterococcus*. The latter taxon had a negative correlation with *Acinetobacter, Stenotrophomonas,* and *Burkholderia*. Interestingly, both *Lactobacillus* and *Enterococcus* were also negatively correlated to *Burkholderia* in tempeh wrapped in plastic.

## Discussion

In the present study, we focused on the differences in bacterial abundance, community structure, and composition from tempeh at the point of sale in Indonesian cities and provinces that were so far not assessed in detail. The results highlighted a substantial impact of the geographical origin on tempeh bacterial abundance, richness, diversity, and community structures.

Significant region-specific patterns in the microbiome of tempeh in Indonesia was observed. These results could be explained by local variations in production techniques. For instance, before fermentation, soybeans are cooked by boiling, either once or twice, to eliminate microbial contaminants (Ahnan‐Winarno et al., 2021). The cooking process does not completely eliminate all members of the indigenous food microbiota but substantially changes the bacterial diversity and community structures (Wicaksono et al., 2022). Tempeh producers in cities in the Central Java province i.e., Pekalongan and Purwokerto, normally employ only one boiling process. Interestingly, the highest bacterial abundance, richness, and diversity were identified in tempeh samples that were obtained from Kebumen city in Central Java province. Therefore, we argue that the initial cooking process could be one critical factor explaining the variations in bacterial abundance, richness, diversity, and community structures. However, neither the cooking procedure nor other production methods were assessed in this study.

One interesting observation found in this study was the effect of geographical distance on bacterial community structures. We found that samples that were obtained from cities in close proximity tend to have a similar bacterial community. Louw (et al., 2023) suggested that fermented food products in specific areas may have distinctive characteristics if some microbial species that contribute distinctive flavor or other attributes of the final fermented products are restricted in their dispersal at large spatial scales. The study involved the collection of tempeh samples at the point of purchase. It is recognized that the sourcing of raw materials, fermentation parameters, and variations in production techniques may influence the bacterial community structures of the final product. However, due to the lack of comprehensive data on this subject, the potential impact of these factors could not be thoroughly investigated. Although there is a possibility that the bacterial community composition may also be influenced by the above-mentioend factors, we observed a significant impact of geographical distance on the structures of bacterial communities. In the wine industry, it is widely recognized that various environmental factors, including climate and biotic factors elements such as soil, grape variety, and fauna, along with viticulture and enological techniques, collaborate to define the sensory characteristics of wine from a specific region, known as terroir (Belda et al., 2017; Bokulich et al., 2016; Knight et al., 2015). The specific microbiota was identified as major key player in translating environmental factors into a specific taste and smell because of their rich portfolio of volatile organic compounds (Verginer et al., 2010). This rationalization is also likely true for tempeh microbiome because different characteristic of tempeh that were obtained from different regions (Kadar et al., 2018). The majority of tempeh is manufactured by cottage industries or home industries, with only a limited number of medium-scale enterprises involved in its production. Currently, the production of tempeh is not subject to strict control, although there are standardization documents accessible. We argue that differences in bacterial community structures from closely distance location might have also been influenced by a similar exposure to individual regions i.e., water and local raw materials and climate. Therefore, the preservation of the natural environment (including soil, water, air, etc.) is crucial for the process of spontaneous fermentation, which holds significant importance for local consumers.

Our study suggests the influence of packaging materials on the enterobacterial abundance but not total bacteria and lactobacilli abundance in final tempeh products. A previous study indicated that the banana leaf serves as a natural habitat for enterobacteriaceae (Köberl et al., 2021, 2015). In the present study, the number of ASVs that belong to *Enterobacteriaceae* was higher in tempeh wrapped in banana leaves in comparison to tempeh wrapped in plastic and the number of ASVs correlated to enterobacterial abundance in tempeh samples (Supplementary Figure S3). We propose that packaging with banana leaves may result in the transfer of plant-associated bacteria to the tempeh products. Moreover, we also suggest that variations in the oxygen permeability of different packaging might have an impact on the growth of facultative anaerobic enterobacteria. The exchange of oxygen is less likely to occur in plastics than in leaves (Owens et al., 2015). This condition likely promotes the growth of enterobacteria in final tempeh products. Previous studies indicated that some bacterial taxa that belonged to *Enterobacteriaceae* such as *Klebsiella* and *Citrobacter* are responsible for the production of vitamin B complex in tempeh (Denter and Bisping, 1994; Keuth and Bisping, 1994). This study emphasizes the significance of banana leaves in terms of their dual role as eco-friendly and sustainable food packaging, as well as their potential to enhance positive microorganism growth in the product.

The tempeh microbiome is dominated by lactobacilli and enterobacteria. Lactic acid bacteria are an integral component of tempeh microbiome. Lactobacilli were suggested to have originated from soybeans which then multiply during the soaking process and acidifying the soybeans (Nurdini et al., 2015). These microbes are likely affected by different abiotic stresses including changes in pH, salinity, temperature, or moisture alongside biotic stresses such as competition and invasion resistance both at the individual or community levels. Lactic acid bacteria are known to harbor unique genes that enable them to digest a variety of plant-derived substances which result in changes in antioxidant activity, and phenolic or vitamin content of fermented food products (Filannino et al., 2018; Kwaw et al., 2018). Moreover, using a correlation network analysis, we found a negative correlation between lactobacilli i.e., *Lactobacillus* and *Enterococcus* with potential unwanted taxa i.e., *Stenotrophomonas, Acinetobacter*, and *Burkholderia*. Those genera have previously been identified as potential opportunistic pathogens and producers of toxic compounds in other fermented foods (Guan et al., 2020; Liu et al., 2019; Moebius et al., 2012; Nahidul-Islam et al., 2018; Rohm et al., 2010). This finding highlights the significance of lactobacilli strains in inhibiting the growth of harmful microorganisms. We propose that the interaction between these microorganisms plays a significant role in determining the quality and safety of tempeh. Although certain ASVs and sequences of isolated strains showed close similarity to sequences of known pathogens, it should be noted that high-throughput amplicon sequencing alone does not provide sufficient evidence to ascertain their pathogenicity or beneficial properties. Further studies combining culturomics and shotgun metagenomics are needed to investigate the potential implication of different microbial community structures and their health-promoting components in tempeh.

In conclusion, we found that the tempeh microbial community was site-specific. The bacterial community structures may have been influenced by local factors such as regional water, raw materials, and climate as well as environmental microbiome. In contrast, packaging materials only had a significant effect on enterobacterial abundance. This study serves as a foundation for further analysis of the functional implications of the tempeh microbiome, which is currently not widely explored. Tempeh, as an integral part of Indonesian cuisine, may also serve as a potential model to investigate the beneficial values of traditional fermented food microbiomes.

## Ethics approval and consent to participate

Not applicable.

## CRediT authorship contribution statement

**Wisnu Adi Wicaksono:** Project administration, Conceptualization, Data curation, Investigation, Methodology, Supervision, Writing – review & editing. **Oluwakemi Elizabeth Akinyemi:** Data curation, Investigation, Methodology, Writing – original draft. **Birgit Wassermann:** Data curation, Investigation, Writing – review & editing. **Samuel Bickel:** Data curation, Investigation, Writing – review & editing. **Antonius Suwanto:** Conceptualization, Resources, Validation, Writing – review & editing. **Gabriele Berg:** Conceptualization, Resources, Validation, Writing – review & editing.

## Declaration of competing interest

The authors declare that they have no known competing financial interests or personal relationships that could have appeared to influence the work reported in this paper.

## Data Availability

The sequencing data has been deposited in the European Nucleotide Archive (ENA) database under the study number PRJEB75498

## Appendix Supplementary materials

Image, application 1
