# Supplementary material for: The terroir of Tempeh: Strong region-specific signatures in the bacterial community structures across Indonesia
Source: Curr Res Microb Sci. 2024 Oct 10;7:100287. doi: 10.1016/j.crmicr.2024.100287 (PMC11533015; doi:10.1016/j.crmicr.2024.100287)
Supplement: Supplementary file 1 [file mmc1.docx]

**The Terroir of Tempeh: strong region-specific signatures in the bacterial community structures across Indonesia**

**Wisnu Adi Wicaksono^a#^, Oluwakemi Elizabeth Akinyemi^a^, Birgit Wassermann^a^, Samuel Bickel^a^, Antonius Suwanto^b^, Gabriele Berg^acd#^**

*^a^Institute of Environmental Biotechnology, Graz University of Technology, Graz, Austria*

*^b^Department of Biology, Faculty of Mathematics and Natural Science, IPB University, Bogor, Indonesia*

*^c^Leibniz-Institute for Agricultural Engineering and Bioeconomy Potsdam (ATB), Potsdam, Germany*

*^d^Institute for Biochemistry and Biology, University of Potsdam, Potsdam, Germany*

^#^Corresponding authors:

Wisnu Adi Wicaksono and Gabriele Berg, Graz University of Technology, Graz

Emails: [wisnu.wicaksono@tugraz.at](mailto:wisnu.wicaksono@tugraz.at) & [gabriele.berg@tugraz.at](mailto:gabriele.berg@tugraz.at)

**Supplementary Materials**

**Supplementary Table S1 Detail of sampling location and packaging materials**

| SampleID | Market | City | Province | Packaging |
| --- | --- | --- | --- | --- |
| TEMP1 | Pasar Baru | Bandung | West Java | Leaf |
| TEMP2 | Kosambi | Bandung | West Java | Leaf |
| TEMP3 | Cihampit | Bandung | West Java | Leaf |
| TEMP4 | Cihampit | Bandung | West Java | Leaf |
| TEMP5 | Cihampit | Bandung | West Java | Leaf |
| TEMP6 | Pasar Baru | Bandung | West Java | Leaf |
| TEMP7 | Pasar Munjul | Jakarta | DKI Jakarta | Leaf |
| TEMP8 | Pasar Cibubur | Jakarta | DKI Jakarta | Leaf |
| TEMP9 | Pasar Kurawasan | Kebumen | Central Java | Leaf |
| TEMP10 | Pasar Mergosono | Kebumen | Central Java | Leaf |
| TEMP11 | Pasar Banyumudal | Kebumen | Central Java | Leaf |
| TEMP12 | Pasar Purwogondo | Kebumen | Central Java | Leaf |
| TEMP13 | Pasar Purbowangi | Kebumen | Central Java | Leaf |
| TEMP14 | Pasar Kolombo | Sleman | Jogjakarta | Leaf |
| TEMP15 | Pasar Kolombo | Sleman | Jogjakarta | Leaf |
| TEMP16 | Pasar Kolombo | Sleman | Jogjakarta | Leaf |
| TEMP17 | Pasar Rejodan | Sleman | Jogjakarta | Leaf |
| TEMP18 | Pasar Rejodan | Sleman | Jogjakarta | Leaf |
| TEMP19 | Kosambi | Bandung | West Java | Plastic |
| TEMP20 | Pasar Baru | Bandung | West Java | Plastic |
| TEMP21 | Pasar Baru | Bandung | West Java | Plastic |
| TEMP22 | Kosambi | Bandung | West Java | Plastic |
| TEMP23 | Cihampit | Bandung | West Java | Plastic |
| TEMP24 | Cihampit | Bandung | West Java | Plastic |
| TEMP25 | Pasar Tugu | Jakarta | DKI Jakarta | Plastic |
| TEMP26 | Pasar Ciracas | Jakarta | DKI Jakarta | Plastic |
| TEMP27 | Pasar Ciracas | Jakarta | DKI Jakarta | Plastic |
| TEMP28 | Pasar Cibubur | Jakarta | DKI Jakarta | Plastic |
| TEMP29 | Pasar Kolombo | Sleman | Jogjakarta | Plastic |
| TEMP30 | Pasar Kolombo | Sleman | Jogjakarta | Plastic |
| TEMP31 | Pasar Kolombo | Sleman | Jogjakarta | Plastic |
| TEMP32 | Pasar Kolombo | Sleman | Jogjakarta | Leaf |
| TEMP33 | Pasar Pemecutan | Denpasar | Bali | Plastic |
| TEMP34 | Pasar Pemecutan | Denpasar | Bali | Plastic |
| TEMP35 | Pasar Kerobokan | Denpasar | Bali | Plastic |
| TEMP36 | Pasar Kerobokan | Denpasar | Bali | Plastic |
| TEMP37 | Pasar Kerobokan | Denpasar | Bali | Plastic |
| TEMP38 | Pasar Anyar | Singaraja | Bali | Plastic |
| TEMP39 | Pasar Buleleng | Singaraja | Bali | Plastic |
| TEMP40 | Pasar Buleleng | Singaraja | Bali | Plastic |
| TEMP41 | Pasar Anyar | Singaraja | Bali | Plastic |
| TEMP42 | Pasar Anyar | Singaraja | Bali | Plastic |
| TEMP43 | Pasar Anyar | Singaraja | Bali | Plastic |
| TEMP44 | Pasar Anyar | Singaraja | Bali | Plastic |
| TEMP45 | Pasar Buleleng | Singaraja | Bali | Plastic |
| TEMP46 | Pasar Buleleng | Singaraja | Bali | Plastic |
| TEMP47 | Pasar Buleleng | Singaraja | Bali | Plastic |
| TEMP48 | Pasar Buleleng | Singaraja | Bali | Plastic |


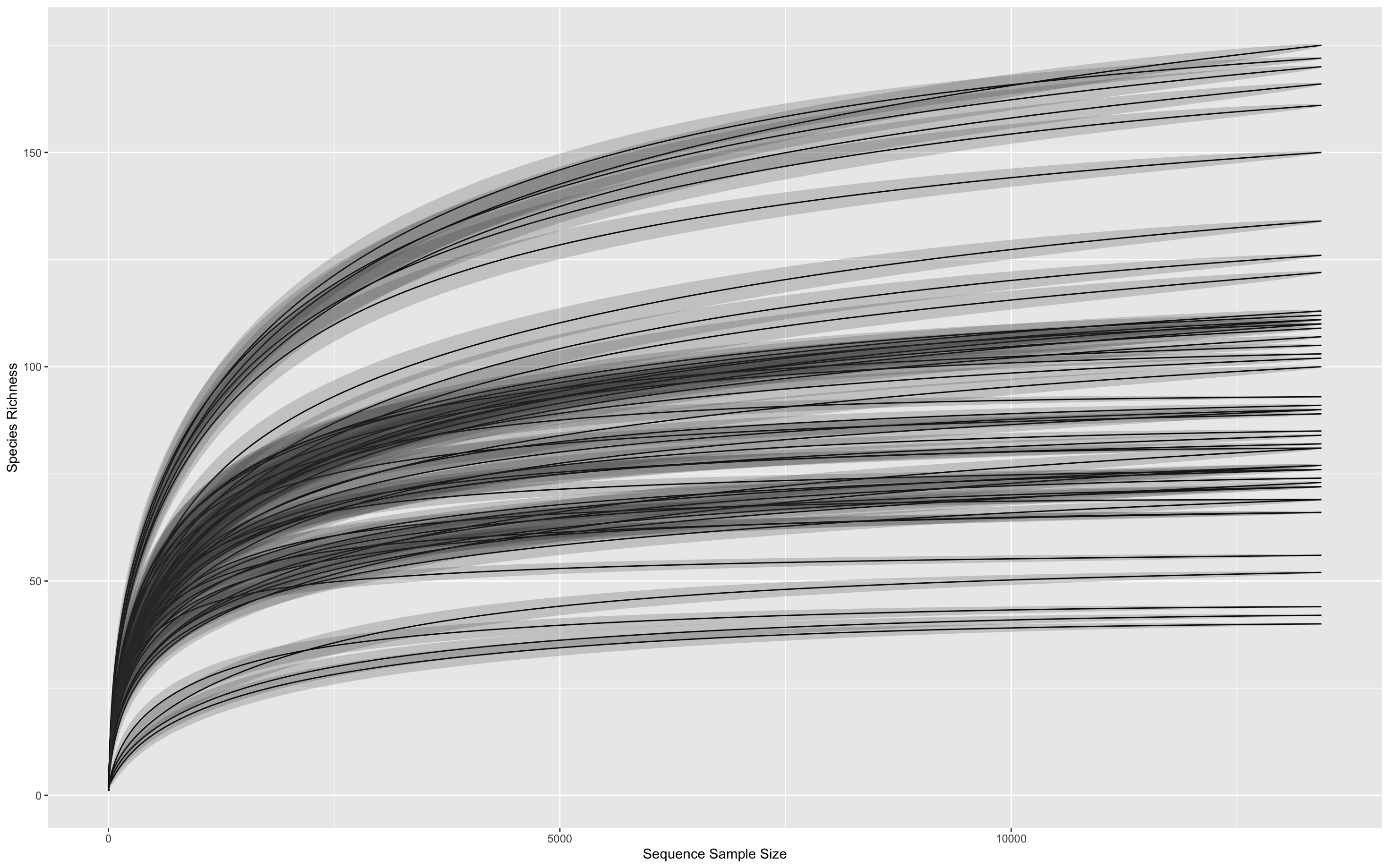


**Supplementary Figure S1 Rarefaction curves showing the number of reads that were classified as bacterial sequences from the rarefied dataset.**


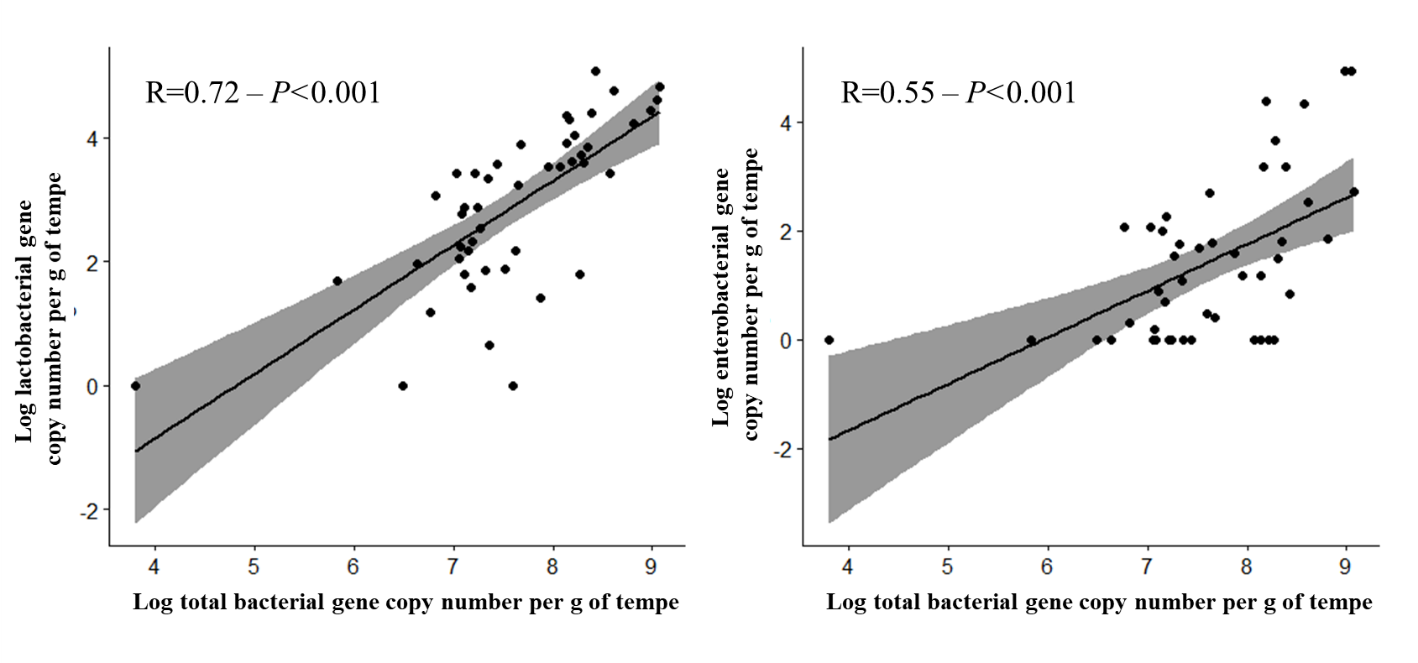


**Supplementary Figure S2 Correlation analysis between lactobacterial abundance and total bacterial abundance (A) and enterobacterial abundance and total bacterial abundance (B).**


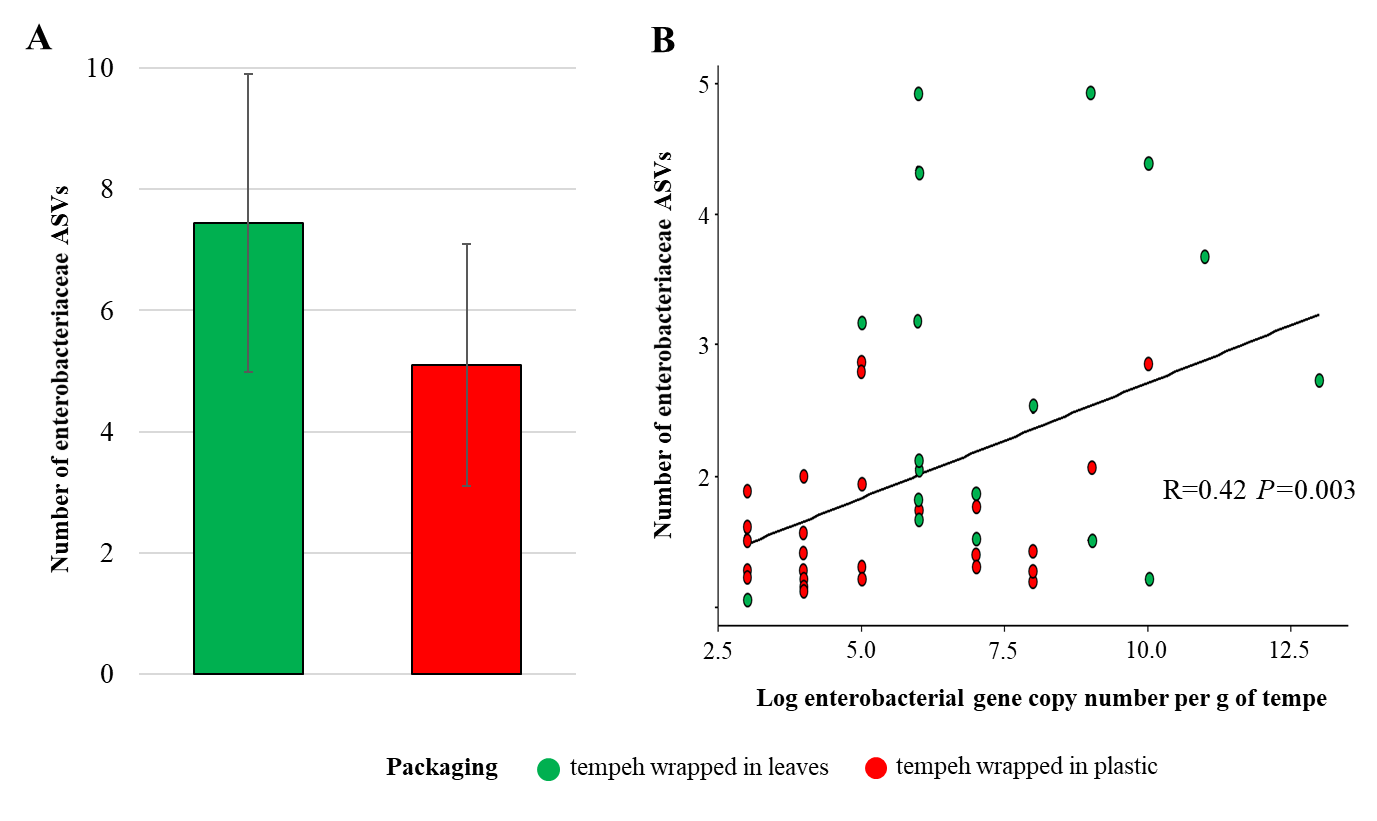


**Supplementary Figure S3 Number ASVs that belong to *Enterobacteriaceae* (A) and correlation analysis between number ASVs that belong to enterobacteriaceae abundance and enterobacterial abundance (B).**
